# Long-term immune checkpoint inhibitor therapy in a patient with metastatic nasopharyngeal carcinoma: a case report

**DOI:** 10.3389/fimmu.2025.1585844

**Published:** 2025-06-12

**Authors:** Defeng Qing, Zhiping Lu, Heming Lu

**Affiliations:** ^1^ Department of Radiation Oncology II, Clinical Oncology Center, People’s Hospital of Guangxi Zhuang Autonomous Region, Nanning, China; ^2^ Faculty of Medical Science, Jinan University, Guangzhou, China

**Keywords:** immune checkpoint inhibitor, immunotherapy, therapy discontinuation, treatment duration, EBV DNA

## Abstract

**Background:**

Immunotherapy has revolutionized cancer treatment. However, the duration of treatment and the timing of discontinuation are major concerns. Current pivotal trials predominantly advocate for a fixed two-year regimen of immune checkpoint inhibitors (ICIs), exemplified by pembrolizumab and toripalimab, as first-line therapy for patients with advanced malignancies. Alternatively, for specific ICIs, including nivolumab, camrelizumab, and tislelizumab, continuous administration until disease progression has emerged as a favored approach. Nevertheless, whether to discontinue treatment after two years remains intensely debated within the medical community, underscoring the need for further research to clarify optimal treatment durations.

**Case presentation:**

In November 2018, a 44-year-old male presented with a persistent headache. Following a positive nasopharyngeal mucosal biopsy, he was diagnosed with non-keratinizing undifferentiated carcinoma of the nasopharynx cT4N2M0. An Epstein-Barr Virus (EBV) DNA load of 800 copies/mL was detected. The patient completed two cycles of induction chemotherapy with liposomal paclitaxel and nedaplatin, followed by platinum-based concurrent chemoradiotherapy, resulting in a progression-free survival (PFS) of 23.6 months. The EBV DNA load dropped significantly to 190 copies/mL. However, during a routine examination in January 2021, metastases in the lung and mediastinal lymph nodes were detected, and the EBV DNA load was measured at 2200 copies/mL. Consequently, surgical intervention was performed, followed by radiotherapy and two years of ICI treatment. Throughout the ICI maintenance period, the EBV DNA level remained consistently below the limit of detection. Remarkably, three months after treatment discontinuation, the patient exhibited a rebound in EBV DNA (1620 copies/mL). Nevertheless, imaging scans revealed no evidence of tumor progression. Following an ICI rechallenge, the patient’s EBV DNA load returned to undetectable levels. The patient continues the ICI therapy and has thus far achieved a PFS of 41.6 months.

**Conclusion:**

EBV DNA levels could serve as an informative marker to predict the necessity of therapy discontinuation during immunotherapy maintenance. Notably, a post-discontinuation ICI rechallenge can still yield favorable outcomes potentially accredited to immune memory.

## Introduction

Immunotherapy represents a pivotal advancement in the treatment of patients with advanced malignant tumors, significantly enhancing outcomes (1). The determination of the optimal duration for immunotherapy remains a contentious issue within the research community. Historically, clinical trials evaluated the efficacy of ICIs for a maximum of two years in responsive patients. For instance, the KEYNOTE-010 trial observed that of the 79 patients who completed a fixed two-year course of ICI therapy, 57.7% maintained PFS at the two-year mark (2). In a related study, the KEYNOTE-024 trial reported that among 39 patients administered pembrolizumab for two years, 82% were still alive after five years (3). The JUPITER-02, CAPTAIN-1st and RATIONALE-309 trials demonstrated that, for the first-line treatment of recurrent or metastatic nasopharyngeal carcinoma (NPC), patients who received ICIs in combination with chemotherapy experienced significantly prolonged PFS and overall survival (OS) compared to those treated with chemotherapy alone. In the JUPITER-02 trial, toripalimab was administered for up to two years. The median PFS was 21.4 months, while the median OS was not reached following a 36-month follow-up period in the toripalimab-based combination group (4). In contrast, in the CAPTAIN-1st and RATIONALE-309 trials, treatment with camrelizumab and tislelizumab persisted until radiographic progression or unacceptable toxicity manifested. In the CAPTAIN-1st trial, the camrelizumab group had a significantly longer PFS than the placebo group, with median PFS of 9.7 months and 6.9 months, respectively (5).Similarly, the RATIONALE-309 trial showed that the tislelizumab group exhibited a markedly longer PFS than the placebo group, with median PFS reaching 9.2 months and 7.4 months, respectively (6). These findings underscore the efficacy of these ICIs in enhancing PFS for the relevant patient populations. To optimize the effectiveness of ICIs, oncologists may prefer to maintain treatment until disease progression or toxicity arises. However, it is currently unclear whether prolonged ICI treatment results in longer survival times. Additionally, oncologists should also take into account the economic burden and adverse events associated with long-term treatment. Balancing the risks and benefits remains a challenging issue in clinical practice.

There is a notable scarcity of data regarding patients with advanced malignancies who have undergone ICI therapy for more than two years, as well as on drug-off criteria in real-world practice so far. Masatoshi Kudo suggested that continuous normalization of three tumor markers (AFP, AFP-L3, and PIVKA-II) for 12 to 24 weeks could serve as a criterion for discontinuing treatment in liver cancer patients with complete responses (7). Additionally, Zhang et al. noted the potential role of circulating tumor DNA (ctDNA) as a meaningful biomarker for assessing whether to continue treatment in advanced cancers (8). For recurrent or metastatic NPC, EBV DNA has been identified as a key prognostic biomarker, primarily serving to evaluate treatment efficacy and monitor disease progression (9–11). Wang et al. found that patients with a ≥ 50% decrease in plasma EBV DNA load at week 4 had an objective response rate of 48.3%, compared to 5.7% for those with a < 50% decrease (12). However, this study only analyzed the association between EBV DNA and treatment response, not treatment discontinuation criteria. Encouragingly, Liu et al. developed an initial prognostic risk stratification model integrating IL-6 and EBV DNA load to predict outcomes in recurrent or metastatic NPC patients treated with ICIs (13). This model may potentially inform future discontinuation criteria. Overall, the criteria for treatment discontinuation in patients with recurrent or metastatic NPC remain poorly validated, leaving a significant gap in real-world data on post-discontinuation relapse rates.

## Case report

**Figure 1:**
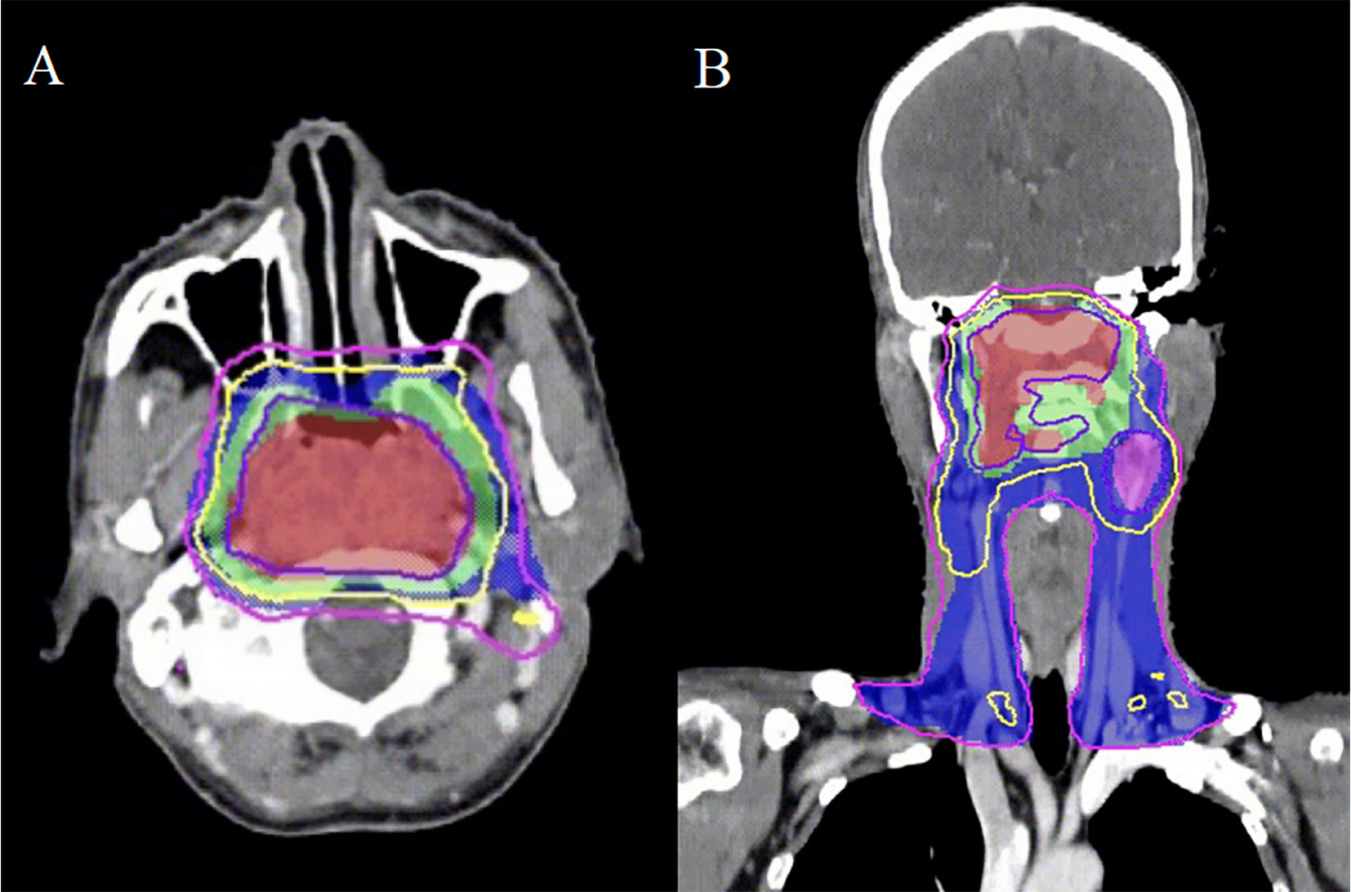
The intensity-modulated radiotherapy plan for nasopharyngeal carcinoma demonstrates three distinct isodose distributions: the gross tumor volume (GTVnx) is encompassed by the 69.96 Gy isodose curve (slate blue), while the clinical target volumes CTV1 and CTV2 are covered by the 60.06 Gy (yellow) and 54.12 Gy (purple) isodose lines, respectively. Transverse **(A)** and coronal **(B)** sections illustrate the treatment plan in these planes.

A 44-year-old male was referred to our institution with a persistent headache in November 2018. The patient had an Eastern Cooperative Oncology Group (ECOG) performance status of 0. Following diagnosis with non-keratinizing undifferentiated carcinoma of the nasopharynx, the patient was staged as cT4N2M0 according to the AJCC 8th edition guidelines. An EBV DNA load amounting to 800 copies/mL was detected. Subsequently, the patient received two cycles of induction chemotherapy with liposomal paclitaxel (135 mg/m² on day 1) and nedaplatin (80 mg/m² on day 1, every 21 days), followed by intensity-modulated radiation therapy (IMRT) combined with concurrent chemotherapy (nedaplatin 100 mg/m² on day 1, every 21 days). The overall treatment course resulted in a PFS of 23.6 months. Radiation dosages administered included: 69.96 Gy in 33 fractions to the gross tumor volume (GTVnx) and to the positive neck lymph nodes (GTVnd), 60.06 Gy to the high-risk clinical target volume (CTV1), and 54.12 Gy to the low-risk clinical target volume (CTV2) (**Figures 1A, B**). The EBV DNA load demonstrated a significant reduction, falling to 190 copies/mL, a finding that underscores the observed efficacy of the treatment.

**Figure 2:**
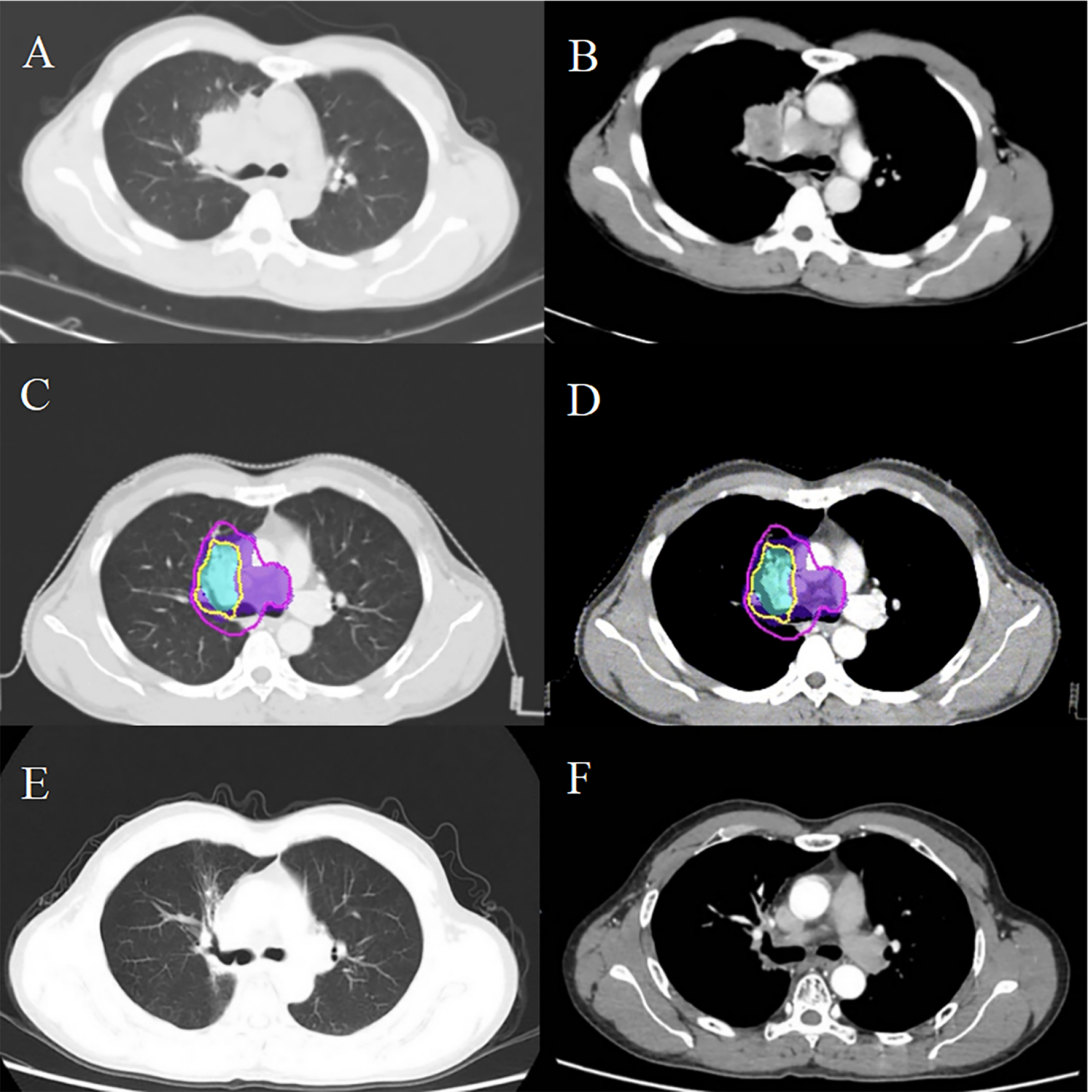
Comparative imaging analysis delineates post-radiotherapy metastatic progression in thoracic regions. Post-treatment surveillance CT identified metastatic lesions in the pulmonary parenchyma **(A)** and mediastinal nodal stations **(B)**. Dosimetric mapping demonstrates therapeutic coverage with the 60 Gy isodose contour (yellow) delineating the GTV, while the 54 Gy isodose (purple) demarcates the CTV in postoperative imaging series **(C, D)**. Serial follow-up imaging shows sustained locoregional control **(E, F)**, with no radiographic evidence of disease recurrence.

**Figure 3:**
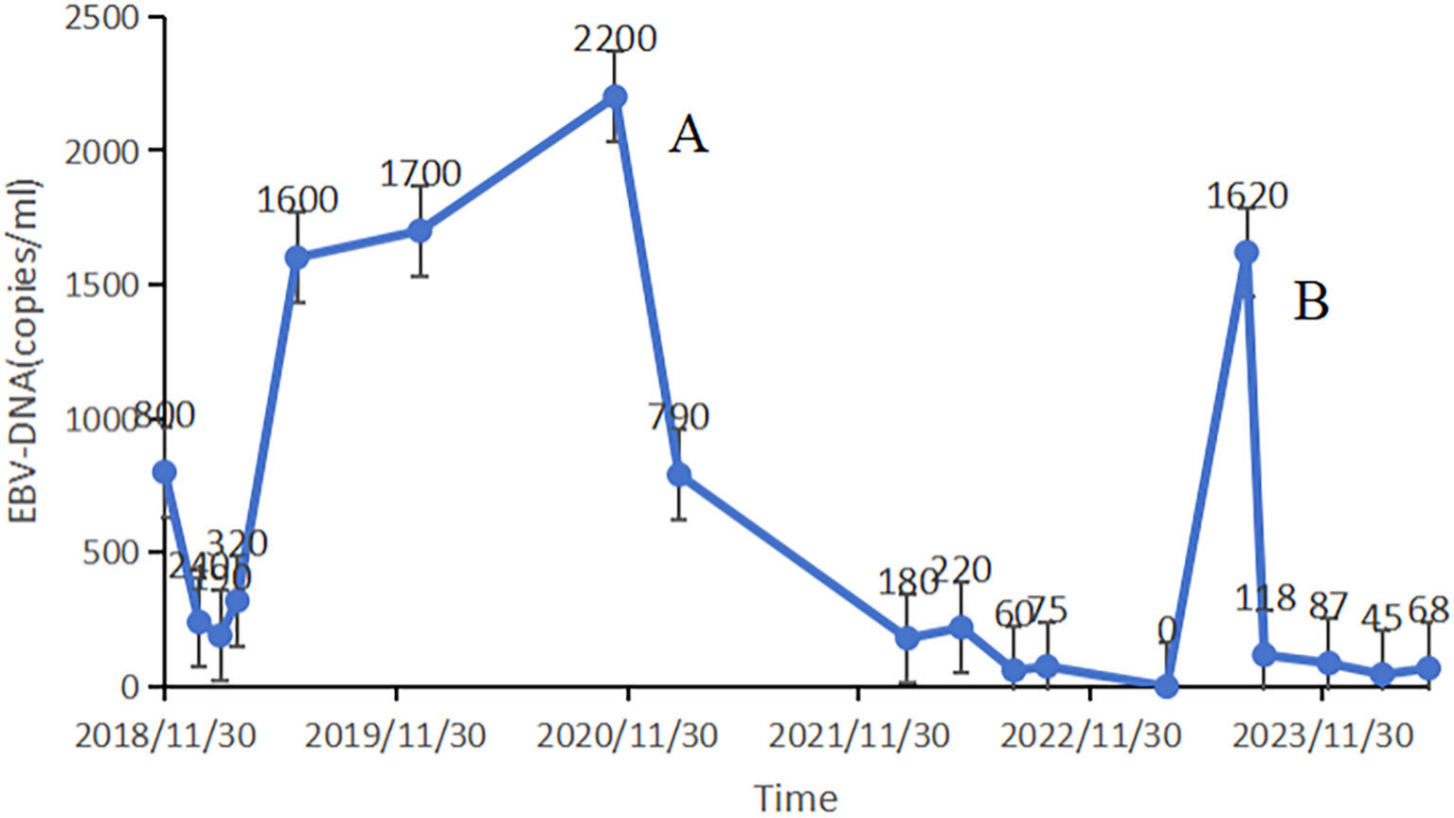
EBV DNA load demonstrates dynamic correlation with disease progression, serving both as a quantitative biomarker for therapeutic monitoring and a predictive indicator for treatment discontinuation criteria. The patient was found to have pulmonary and mediastinal lymph nodes metastases **(A)**. A rebound in EBV DNA levels was recorded three months after discontinuing the treatment **(B)**.

In January 2021, routine examination revealed metastases in the patient’s lung and mediastinal lymph nodes (**Figures 2A, B**). Concurrently, the EBV DNA load was found to be 2200 copies/mL. Subsequently, he underwent wedge resection and lymph node biopsy. Immunohistochemistry showed the tumors were positive for PD-L1. First-line treatment was initiated with GP (gemcitabine 1000 mg/m² on days 1 and 8, cisplatin 75 mg/m² on day 1, every 21 days) and toripalimab (240 mg on day 1, every 21 days) for one course. During treatment, the patient experienced grade 4 neutropenia, leading to chemotherapy suspension due to intolerable toxicity. After recovery from myelosuppression, radiotherapy was administered, with prescription doses of 60 Gy in 30 fractions to the GTV and 54 Gy to the CTV via IMRT (**Figures 2C, D**). The patient received two years of toripalimab maintenance therapy after IMRT, which induced grade 2 hypothyroidism managed with thyroxine hormone replacement therapy. Throughout the ICI maintenance period, EBV DNA load remained persistently below the detectable threshold. Three months after treatment cessation, an EBV DNA rebound was documented, peaking at 1620 copies/mL. However, imaging scans revealed no tumor progression (**Figure 2E, F**). Following an ICI rechallenge, the patient’s EBV DNA load returned to undetectable levels (**Figure 3**). The patient continues ICI treatment and has achieved a PFS of 41.6 months thus far.

## Discussion

The optimal duration of immunotherapy, whether finite or continuing until progression, remains a prominent subject of debate. While the initial phase I trials proposed a 2-year limit to therapy (14, 15), several previous clinical trials have demonstrated that patients who completed two years of ICI treatment experienced long-term PFS and OS (4, 16, 17). For instance, the KEYNOTE-010 trial revealed that patients treated with pembrolizumab for two years exhibited favorable prognoses, with 1-year PFS and OS rates of 72.5% and 98.7%, respectively (16). Similarly, In the JUPITER-02 trial, patients with recurrent or metastatic NPC showed clinically significant PFS and OS benefits after two years of toripalimab treatment. ICI led to a remarkable extension of PFS, with a median PFS of 21.4 months for the toripalimab group versus 8.2 months for the placebo group. Furthermore, the median OS in the ICI group was not reached at the time of analysis, while it was 33.7 months in the placebo group, highlighting the substantial survival benefit of toripalimab-based combination therapy (4). The findings from both trials provide substantial evidence in support of ICI as an effective first-line treatment strategy for recurrent or metastatic NPC.

A retrospective analysis of patients with advanced non-small cell lung cancer (NSCLC) treated with immunotherapy found no significant impact on patient OS after treatment discontinuation at two years (18). This analysis evaluated 706 patients and showed that discontinuing immunotherapy at two years versus continuous treatment was associated with OS rates of 79% and 81%, respectively, with no significant difference in mortality risk, indicating that discontinuation may be safe (18). In contrast, the CheckMate-153 trial demonstrated longer median PFS (24.7 months vs. 9.4 months) and OS (not reached vs. 28.8 months) in patients receiving continuous versus one-year fixed-duration treatment, suggesting that continuing immunotherapy may improve outcomes (19). Additionally, the KCSG LU20–11 study reported that the majority of patients who discontinued ICI after two years experienced disease progression within the first 12 months (20). Together, these findings highlight the dilemma faced by oncologists in balancing treatment continuation with clinical benefit. The prolonged ICI maintenance duration [24 months vs. the median 7–9 months reported in KEYNOTE-048 (21)] underscores the critical need for disease-specific treatment guidelines to inform optimal discontinuation strategies.

No definitive guidelines govern the discontinuation of immunotherapy in patients exhibiting an objective response. While Kudo’s recommendations provide clinicians with a framework for individualized therapeutic decision-making regarding safe treatment discontinuation in hepatocellular carcinoma (7), scarce research has explored similar scenarios after immunotherapy discontinuation in head and neck squamous cell carcinoma (HNSCC). Although KEYNOTE-048 validated pembrolizumab in PD-L1-high HNSCC, our patient with EBV-driven NPC required a tailored approach, prioritizing EBV DNA monitoring over PD-L1 status. Monitoring EBV DNA load—a key biomarker for diagnosis, treatment response, and potential immunotherapy management in nasopharyngeal cancer—is pivotal. A retrospective analysis showed EBV DNA had 85.9% and 92.8% accuracy in detecting regional recurrence and distant metastasis, respectively (22). The POLARIS-02 study showed that 14 patients responding to toripalimab had at least a 100% increase in EBV DNA titer 3 months before radiographic disease progression (12). Moreover, an analysis demonstrated that 40% of patients with complete or partial response experienced significantly increased EBV DNA load during ICI maintenance, leading to disease progression. All patients with stable disease had significantly elevated EBV DNA load during this period, and 74.2% subsequently developed progression (23). These findings suggest that substantial EBV DNA load increases during ICI maintenance may serve as an early predictor of progression and a potential indicator for continuing treatment. In our case, EBV DNA load remained within normal ranges for two years during immunotherapy. Three months after ICI discontinuation, viral load rebounded, but returned to undetectable levels after therapy resumption. This may be indicative of a rapid clearance of antibodies, which could result in a relatively brief treatment duration in the local tumor environment (24). It is also possible that resistance mechanisms may develop when chronic PD-1 blockade is removed (25), underscoring the need for prolonged immunotherapy. Improved treatment outcomes generally justify continuing ICI administration.

The report has certain limitations. First, it does not explore biological mechanisms underlying EBV DNA rebound, such as clonal evolution or immune escape, nor validate rebound events through repeat biopsies. Second, the absence of comparisons to analogous cases in the literature hinders interpretation of whether the observed EBV DNA rebound represents a typical or exceptional clinical scenario.

Clinicians must carefully balance the benefits of prolonged ICI therapy against late toxicity risks while integrating multifaceted patient-specific factors. In our case, continuation of treatment despite EBV DNA rebound was justified by the patient’s asymptomatic status and durable radiographic response, reflecting ongoing tumor control. However, broader considerations, including performance status, severity of side effects, financial burdens, and patient preference, are equally critical for tailoring optimal immunotherapy duration and aligning with the patient-centered goals.

## Conclusion

Determining the optimal duration of immunotherapy should be informed by the risk-benefit profile of each individual. Current literature suggests that a two-year course of immunotherapy might be reasonable, but an extended course may also yield survival benefits. Notably, EBV DNA load may serve as a predictive biomarker for guiding therapy discontinuation during immunotherapy maintenance. As several prospective “stop or go” studies on immunotherapy are underway, we await further insights.

## Data Availability

The raw data supporting the conclusions of this article will be made available by the authors, without undue reservation.
